# Incidental adenocarcinoma of the gallbladder in a patient with Y insertion gallbladder duplication in the context of recurrent biliary colic: A video case report

**DOI:** 10.1097/MD.0000000000028829

**Published:** 2022-02-25

**Authors:** Dustin Jefferys, Susmit Roy, Adeeb Majid

**Affiliations:** Department of General Surgery, Calvary Mater Newcastle, Australia.

**Keywords:** adenocarcinoma, case report, gallbladder cancer, gallbladder duplication, laparoscopic cholecystectomy

## Abstract

**Rationale:**

Gallbladder cancer is the most common malignancy of the biliary tree. Despite this, the only curative therapy remains surgical resection of the lesion achieving microscopically clear margins before malignant spread has occurred. Gallbladder duplication is an uncommon anatomical variance which occurs globally. It can present in a range of ways dependent on the embryological origin of the variance.

**Case:**

A 52-year-old female presented for planned laparoscopic cholecystectomy in the context of cholelithiasis resulting in recurrent biliary colic. The patient had no personal history of malignancy or significant medical comorbidities.

**Diagnosis:**

Intraoperatively, the patient was found to have Y-insertion variation of gallbladder duplication. Histopathology of the resected gallbladders showed an incidental invasive gallbladder adenocarcinoma affecting one of the gallbladders.

**Intervention:**

Both gallbladders were laparoscopically resected en-bloc.

**Outcomes:**

The patient underwent oncology staging, which found no evidence of metastatic spread. Regular surveillance is attended with no recurrence of disease identified.

**Conclusion:**

There are few reported cases detailing the occurrence of gallbladder adenocarcinoma in the presence of duplication of the gallbladder. This case demonstrates the clinical benefit of R0 surgical resection of gallbladder cancer, whilst highlighting the difficulties of diagnosing duplication of the gallbladder or gallbladder adenocarcinoma.

## 1 Introduction

Gallbladder (GB) duplication is an uncommon congenital anomaly which occurs in approximately 1 in every 4000 people.^[1–3]^ However, they are not identified unless they become symptomatic with GB pathology such as cholecystitis/cholangitis needing procedures such as cholecystectomy/endoscopic retrograde cholangiopancreatography or incidentally found in imaging or during autopsy. Duplication of the gallbladder is associated with an increased risk of cholecystic complications including cholecystitis and cholelithiasis. There is also an increase in post-surgical complications in these patients.^[3,4]^

Mostly, both are benign and it is very rare to find adenocarcinoma in one of the GB in the case of duplication of GB. Only 5 reported cases of this coincidence have been identified in a recent literature review.^[2,5–8]^

We present a rare case of Adenocarcinoma which was found incidentally during laparoscopic cholecystectomy of a Y-insertion GB duplication.

## 2 Case

Our patient, a 52-year-old woman, underwent an elective laparoscopic cholecystectomy, and intraoperative cholangiogram (IOC) secondary to symptomatic cholelithiasis causing recurrent biliary colic. The patient had no significant history of medical ailments or malignancy. Pre-operative ultrasound imaging showed a thick-walled gallbladder with multiple mobile stones up to 7.3 mm in size and associated biliary sludge. Additional evidence of mild adenomyomatosis was noted.

The procedure was performed under general anaesthesia. Port placements were as for standard laparoscopic cholecystectomy (American way). Intra-operative direct vision of the anatomy revealed duplication of the gallbladder. Two unique gallbladder lobes were present, joined together with a septum. There were 2 separate cystic arteries which were separately ligated and divided. IOC confirmed presence of a single common cystic duct with normal anatomy. It drained in the common hepatic duct to form the common bile duct. The rest of the biliary tree anatomy was normal. There was good flow of contrast both distally and proximally.

With careful dissection of Calot triangle, Strasberg critical view of safety was achieved and cholecystectomy with IOC performed (See Image S1, Supplemental Digital Content, which demonstrates the normal post cholecystectomy anatomy of the bile duct). Complete haemostasis was achieved, and washout performed. The gallbladders were retrieved en-bloc in a single bag through the umbilical port and sent for histopathology.

The histopathology from the resected specimens confirmed the presence of duplicated gallbladder with each lobe joining to form a common cystic duct in Y-configuration. Microscopy revealed a 3 mm focus of invasive adenocarcinoma involving the mucosa and muscularis propria of one of the gallbladders. This was in a background of extensive high-grade dysplasia. The cystic duct margin, sampled lymph nodes, and second gallbladder were negative for dysplasia. The gallbladder neoplasm was staged as a pT1b tumor.

The patient was discussed at our multi-disciplinary team (MDT) meeting and a staging computed tomography (CT) scan of the chest, abdomen, and pelvis was performed. There was no evidence of metastatic disease or pathologic lymph nodes. Tumour markers CEA and CA 19-9 were within normal range.

The patient received no additional treatment for malignancy and attended 3-, 6-, 12-, and 24-month follow-up with no recurrence of disease.

(See Video S1, Supplemental Digital Content, which shows an abridged version of the operation up to the point of IOC. The video demonstrates the intra-operatively identified anatomical variance of gallbladder duplication)

Further review of the patient's pre-operative ultrasound scan images (Image S2, Supplemental Digital Content, which demonstrate the preoperative ultrasound findings) have failed to recognize the intra-operatively identified pathology of gallbladder duplication. This has been verified by 2 independent radiologists accredited with RANZCR.

## 3 Discussion

Gallbladder duplication can present with multiple variants of structure which can be categorized in to 2 embryological origins. It can occur as a result of split primordium arising from a single embryological origin and resulting in a bilobed gallbladder with 2 chambers, or development of an unique accessory gallbladder arising from dual embryological origins.^[9,10]^ This results in the attachment of 2 independent cystic ducts to the biliary bile duct. This can be further categorized using Boyden's classification.^[11,12]^ The presented case demonstrates a ‘Y’ variant gallbladder (See Figure S1, Supplemental Digital Content, which demonstrates the Boyden classification of GB duplication,). This variant is the second most common form seen in duplication of the gallbladder, accounting for roughly 24% of cases. The most common presentation, representing approximately 48% of cases is the “H” or “ductal” variant in which 2 separate gallbladders with unique cystic ducts form and attach independently to the common bile duct. Less common are the septate of bilobed variants in which a single cystic duct is shared. These represent approximately 10% and 9%, respectively.^[13]^

Despite being the most common malignancy of the biliary tree and the third most common cancer of the gastrointestinal tract, cancer of the GB remains a rare neoplasm which displays notable geographic variability.^[14,15]^ It is relatively less prevalent in developed countries like Australia, displaying an approximate incidence of 1.4 cases per 100,000 population.^[16,17]^

GB cancer is more common in females at a ratio exceeding 2:1, has a higher prevalence in Caucasians and has an incidence which increases with age.^[16,18]^ Whilst the overall incidence has decreased in developed countries, the prevalence in younger populations has risen.^[19]^ There are also a number of disease risk factors associated with the development of gallbladder cancer. Many of these share a common characteristic of chronic inflammation of the gallbladder. Gallstones are one of the most common risk factors for the development of gallbladder cancer and are found in up to 90% of cases.^[20]^ Other risk factors include, but are not limited to, chronic infections of the gallbladder, congenital biliary cysts, and obesity. Incidentally, patients with duplication of the gallbladder are more prone to possess a higher incidence of many of these above risk factors.

There are no surveillance programs to detect GB cancer in the general population. In patients with symptoms commensurate with biliary tract pathology, ultrasound (US) is typically the initial imaging modality to identify employed. This is largely due to its accessibility and absence of ionizing radiation when first investigating more benign biliary pathology. However, studies suggest mixed sensitivity of US in the identification of GB carcinoma and poor sensitivity for investigating local spread of disease and additional multi-planar imaging with computed tomography and magnetic resonance imaging is often required.^[21]^ The sensitivity of US can be further complicated in the case of GB duplication where abnormalities of malignancy may be difficult to differentiate from the common anatomical variants associated with the duplication, which can itself be difficult to distinguish from more common pathological changes such as Phrygian cap or choledochal cysts.^[9,22]^ Current laboratory studies largely remain non-sensitive and non-specific for GB cancer. Tumor markers including Carcinoembryonic Antigen or Carbohydrate Antigen 19-9 may be elevated in some cases but are non-specific to the GB. Similarly alkaline phosphatase or serum bilirubin may be elevated but generally only when sufficient disease progression has occurred to facilitate bile duct obstruction.^[23]^

GB cancer has a high mortality rate which is attributed to the nonspecific features of disease, frequent advanced stage at time of diagnosis, and the significance of organs in proximity to the gallbladder's position. Presently, the only curative management of disease is surgical, requiring microscopically margin-negative resection (R0 resection) of disease before peripheral spread has had the opportunity to occur.^[24–26]^

## 4 Conclusion

Gallbladder duplication is an uncommon congenital anomaly which occurs globally. Cancer of the gallbladder is a rare form of malignancy amongst Australian populations but carries a high rate of mortality due to the often-late stage of disease at time of diagnosis. The outcome of this unusual case demonstrates the survival benefit of early identification and full resection of disease with clear margins.

## Acknowledgments

Thank you - Dr Richard Woodford - MBBS, FRANZCR; Radiology, John Hunter Hospital, Newcastle.

## Author contributions

**Conceptualization:** Susmit Roy.

**Data Curation:** Susmit Roy, Dustin Jefferys.

**Resources:** Adeeb Majid, Susmit Roy, Dustin Jefferys.

**Software:** Dustin Jefferys, Susmit Roy.

**Supervision:** Adeeb Majid and Susmit Roy.

**Writing – Original Draft:** Dustin Jefferys.

**Writing – Review and Editing:** Dustin Jefferys, Susmit Roy, Adeeb Majid.

## Supplementary Material

Supplemental Digital Content

## Supplementary Material

Supplemental Digital Content

## Supplementary Material

Supplemental Digital Content

## Supplementary Material

Supplemental Digital Content
