# Supplementary material for: Incidental adenocarcinoma of the gallbladder in a patient with Y insertion gallbladder duplication in the context of recurrent biliary colic: A video case report
Source: Medicine (Baltimore). 2022 Feb 25;101(8):e28829. doi: 10.1097/MD.0000000000028829 (PMC8878699; doi:10.1097/MD.0000000000028829)

**Image 1.** Intra-operative cholangiogram demonstrating normal post cholecystectomy anatomy of the bile duct. **a)** Normal biliary tree. **b)** Normal drainage of contrast via the common bile duct


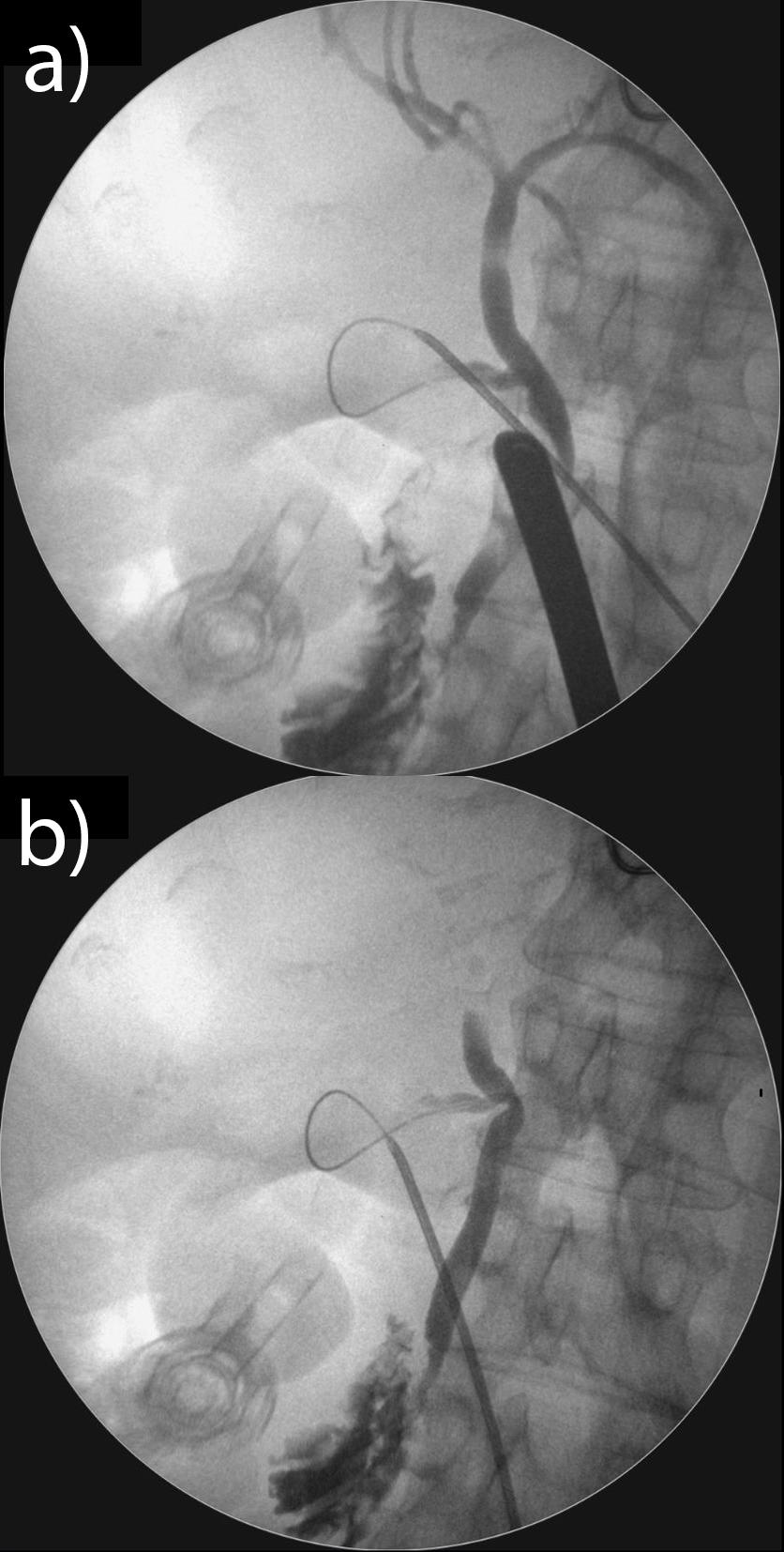

Supplement: Supplemental Digital Content [file medi-101-e28829-s001.doc]
