# Supplementary material for: Incidental adenocarcinoma of the gallbladder in a patient with Y insertion gallbladder duplication in the context of recurrent biliary colic: A video case report
Source: Medicine (Baltimore). 2022 Feb 25;101(8):e28829. doi: 10.1097/MD.0000000000028829 (PMC8878699; doi:10.1097/MD.0000000000028829)

**Image 2.** Pre-operative ultrasound gallbladder suggesting GB sludge, calculi and adenomyomatosis. No evidence of gallbladder duplication. **a)** Gallbladder long view 1 **b)** Gallbladder long view 2 **c)** Gallbladder long view 3


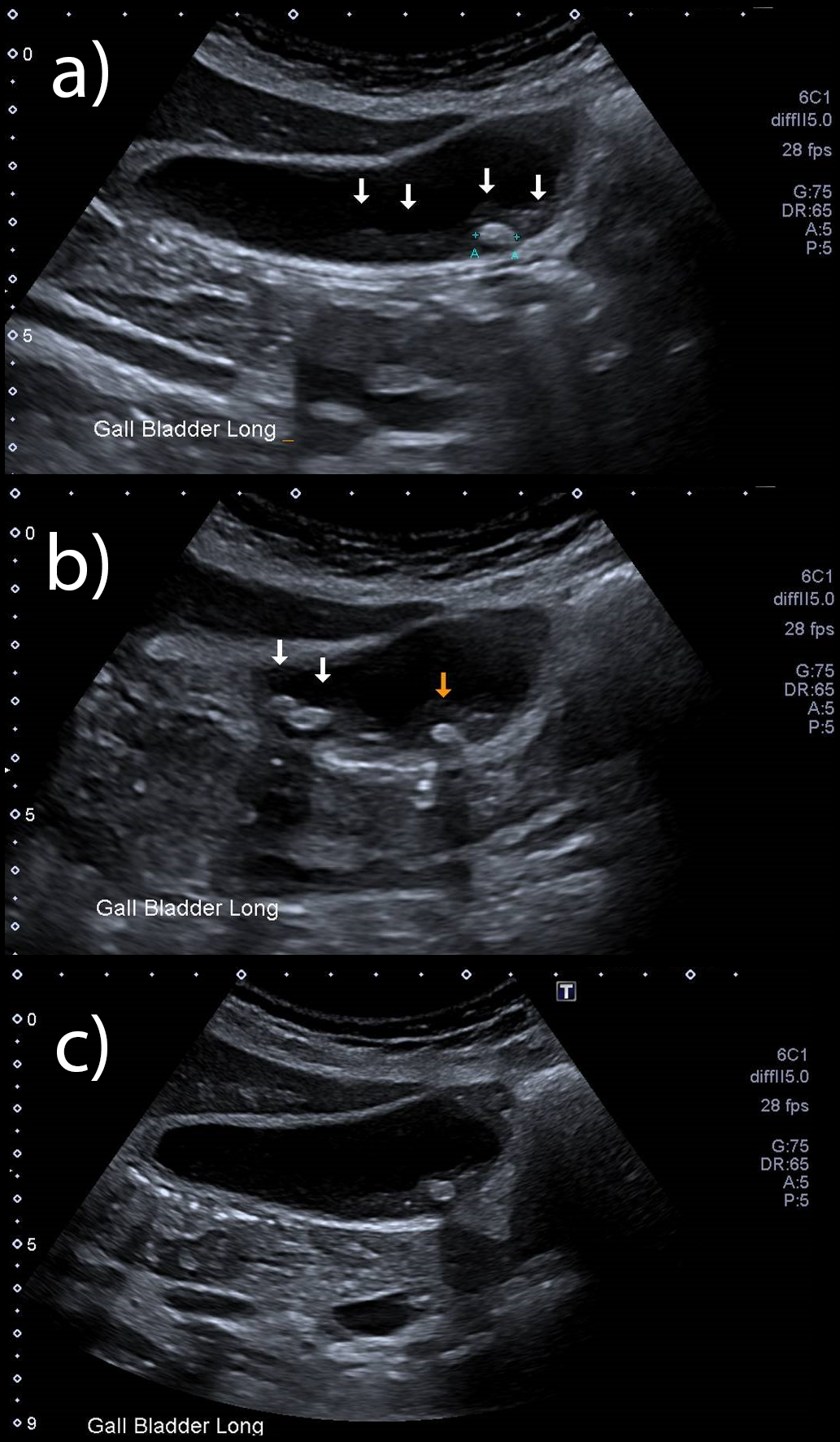

Supplement: Supplemental Digital Content [file medi-101-e28829-s003.doc]
