# Supplementary figures and images for: Incidental adenocarcinoma of the gallbladder in a patient with Y insertion gallbladder duplication in the context of recurrent biliary colic: A video case report
Source: Medicine (Baltimore). 2022 Feb 25;101(8):e28829. doi: 10.1097/MD.0000000000028829 (PMC8878699; doi:10.1097/MD.0000000000028829)

**Figure 1.** Boyden’s classification of gallbladder duplication (13)


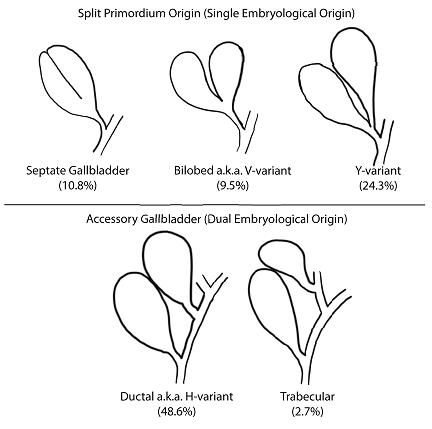

Supplement: Supplemental Digital Content [file medi-101-e28829-s004.doc]
